# *Cordyceps militaris* extract and cordycepin ameliorate LPS-challenged colonic damage in piglets by modulating the microbiota and metabolite profiles

**DOI:** 10.3389/fimmu.2025.1530098

**Published:** 2025-03-10

**Authors:** Shijie Xiong, Fan Wan, Jiajia Jiang, Yanfang Liu, Yiqiong Hang, Huiqin Xue, Yang Lu, Yong Su

**Affiliations:** ^1^ Institute of Animal Husbandry & Veterinary Science, Shanghai Academy of Agricultural Sciences, Shanghai, China; ^2^ Laboratory of Gastrointestinal Microbiology, Jiangsu Key Laboratory of Gastrointestinal Nutrition and Animal Health, College of Animal Science and Technology, Nanjing Agricultural University, Nanjing, China; ^3^ Shanghai Engineering Research Center of Breeding Pig, Shanghai, China; ^4^ Institute of China Black Pig Industry Research, Zhejiang Qinglian Food Co., Ltd., Haiyan, China

**Keywords:** piglets, *Cordyceps militaris* extract, cordycepin, lipopolysaccharide, colon microbiota, metabolomics

## Abstract

**Introduction:**

*Cordyceps militaris* extract (CME) and cordycepin (CPN) are biomolecules with a wide range of biological activities, including anti-inflammatory, antioxidant and anti-tumour effects. The research objective wasto investigate the influences of CME and cordycepin CPN on colonic morphology, microbiota composition and colonic metabolomics in lipopolysaccharide (LPS)-challenged piglets.

**Methods:**

Twenty-four weaned castrated piglets were randomly divided into four groups: control group (fed basal diets), LPS group (fed basal diets), CPN-LPS group (basal diets + 60 mg/kg cordycepin), and CME-LPS group (basal diets + 60 mg/kg *C. militari*sextract). On the 21st day, the LPS, CPN-LPS, and CME-LPS groups received an injection of 100 μg/kg BW LPS, while the control group was given sterile saline.

**Results:**

The findings demonstrated that CPN or CME attenuated intestinal morphology damage with LPS-challenged piglets. CPN and CME alleviated intestinal microbiota dysbiosis and metabolic disorders under LPS-challenged by enriching serum protein levels, regulating of inflammatory cytokine secretion and altering colonic microbial composition. Colonic microbiota analysis that the CPN improved the relative abundance of Acidobacteriota and inhibited *Faecalibacterium*, CME promoted the relative abundance of *Prevotella* and *Lachnospiraceae NK4A136*group. Meanwhile, the alleviation of colonic damage is achieved through modulation of metabolic pathways linked to tryptophan metabolism, biosynthesis of amino acids and butanoate metabolism.

**Discussion:**

Conclusively, our preliminary findings reveal that CPN or CME could serve as a beneficial dietary supplement to alleviate gut diseases in weaning piglets.

## Graphical Abstract

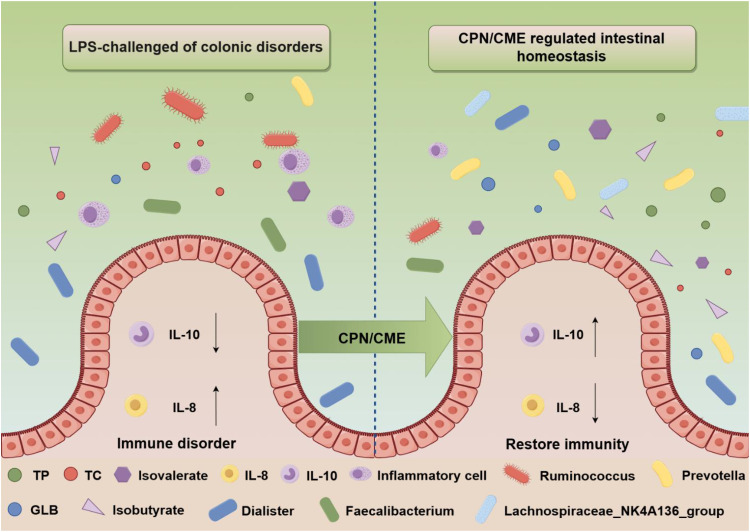

## 1 Introduction

Gut microbiota and its derived metabolites are crucial for preserving the integrity of the gut and maintaining the balance of the intestinal ecosystem. These microorganisms engage with the host and participate in a range of physiological processes, including nutrient metabolism, immunomodulation, and intestinal barrier integrity (1–3). It is well established that variation in the composition of the microbiota can modulate susceptibility to colitis (4). The colonization of the intestinal bacteria and the construct of the intestinal immune system occur during infant stages (5). Hence, a premature departure from maternal nutrition and abrupt changes in the external environment can lead to impaired immune function and gut flora disruption in young animals, ultimately increasing their susceptibility to enteric diseases (6). Nevertheless, intensive farming accelerates the process of weaning piglets. It has already been proven that in the process of early weaning, weaned piglets exhibit a reduction in appetite, lowered immunity, heightened incidence of diarrhea, and, in extreme cases, mortality (7).

In recent years, natural extracts and bioactive substances have shown great potential in improving gut health. For example, *Cymbopogon citratus* and *Poria cocos* polysaccharides can have beneficial effects on the metabolic functions of certain microorganisms, thereby maintaining the health of the organism (8, 9). In addition to these, probiotics and prebiotics are widely studied and applied to improve intestinal health. Probiotics, such as *Lactobacillus* and *Bifidobacterium*, can directly regulate the composition of the gut microbiota and immune response. They can inhibit the growth of harmful bacteria by competing for adhesion sites and nutrients in the intestine and also produce antibacterial substances like bacteriocins (10, 11). Prebiotics, however, can be used as a food source of probiotics, which can promote the growth and activity of beneficial intestinal bacteria, thereby improving the intestinal environment, enhancing nutrient absorption, and reducing inflammation (12).

*Cordyceps militaris*, valued for its medicinal properties, is extensively utilized as a dietary supplement and medicine across Asia (9). *C. militaris* extract (CME) is rich in various bioactive substances, like cordycepin (CPN), carotenoids, and cordyceps acid (13). Among the many valuable components in *C. militaris* extract, cordycepin exhibits a variety of bioactive functions. The bioactive functions of cordycepin involve anticancer, antioxidant, and viral infection inhibition (14, 15). A study demonstrated that cordycepin synergizes with CTLA-4 suppressant to alter the responsive and depletion condition of CD8T cells, thus enhancing CD8 T cell-mediated tumor microenvironment (TME) anti-tumor immunity, remodeling the tumor microenvironment, and enhancing cancer immunotherapy (16). Moreover, cordycepin has been reported to be protective against intestinal inflammation in zebrafish larvae caused by cadmium exposure (17). Numerous animal and clinical trials have demonstrated the multiple health benefits and pharmacological activities of cordyceps extracts. For instance, *C. militaris* extract has been shown to enhance intestinal immunity in growing pigs by modulating the gut barrier function and gut microbiota (18). Research has shown that *C. militaris* extract can reduce the magnitude of Dextran Sulfate Sodium (DSS)-triggered colitis in mice by lowering inflammatory factor levels (19). Our earlier investigations showed that supplementing with CPN or CME suppressed lipopolysaccharide (LPS)-challenged small intestinal injury and suppressed inflammatory responses in weaning piglets. Concomitantly, CPN or CME mitigated inflammation and oxidative stress by modulating the ileum microbiota composition and regulating genes associated with complement activation, which are involved in inflammation and immune reactions (20). However, compared with probiotics and prebiotics, the research on CME and CPN in the field of intestinal health is relatively new. Although probiotics and prebiotics have been widely used and studied, they also have some limitations. Probiotics may face challenges such as poor survival in the harsh intestinal environment and potential side effects in some individuals (21). Prebiotics, while promoting the growth of beneficial bacteria, may also be utilized by some harmful bacteria in certain cases (22). In contrast, CME and CPN have unique advantages. CME and CPN are natural products with multiple bioactive components, which may have more comprehensive effects on intestinal health. Notably, the ileum and colon, as essential components of the intestines, have significant parenchymal morphological and structural variance, as well as differences in their physiological functions and microbial community compositions (23). In particular, mammalian gut microbial composition and function, metabolomics products, and host–microbiota interactions are quite different between different gut locations and gut compartments (24, 25). It remains unclear whether CPN and CME exhibit distinct efficacies in protecting against colonic damage and the specific mechanisms that involve intestinal microecology and metabolic pathways.

In light of the preceding discussion, it is imperative to examine the effect of supplementation with CPN or CME on LPS-challenged colonic microbiota and metabolites in piglets. The purpose of this research was to comprehensively assess the effects of CPN or CME supplementation on colonic health in weaned piglets. Fundamentally, we aimed to investigate the effect of supplementation with CPN or CME on the morphological structure of the LPS-induced colon in piglets. Any change in morphological structure directly affects intestinal functions such as nutrient absorption and barrier function. We then explored how these structural and functional changes interact with the composition of the microbiota in the colon. In turn, the microbiota plays a crucial role in various metabolic processes, so we also investigated the impact of the microbiota on metabolites. Ultimately, by understanding these interrelationships, we can elucidate the specific mechanisms by which supplementation with CPN or CME affects the colon and ileum of LPS-challenged piglets, thereby providing greater insight into their role in maintaining colon health.

## 2 Materials and methods

### 2.1 Preparation of CPN and CME

Preparation for CPN and CME adheres to the established procedures of previous study (20). Briefly, the *C. militaris* residue sample (Kangneng Biological Engineering Co., Ltd., Yang Zhou, China) was milled, water extracted, concentrated, and spray-dried to obtain CME containing 1% cordycepin. CPN, with a 90% purity level, was sourced from Shanghai Yuanye Bio-Technology Co., Ltd., located in Shanghai, China.

### 2.2 Animals and experimental design

**Figure 1:**
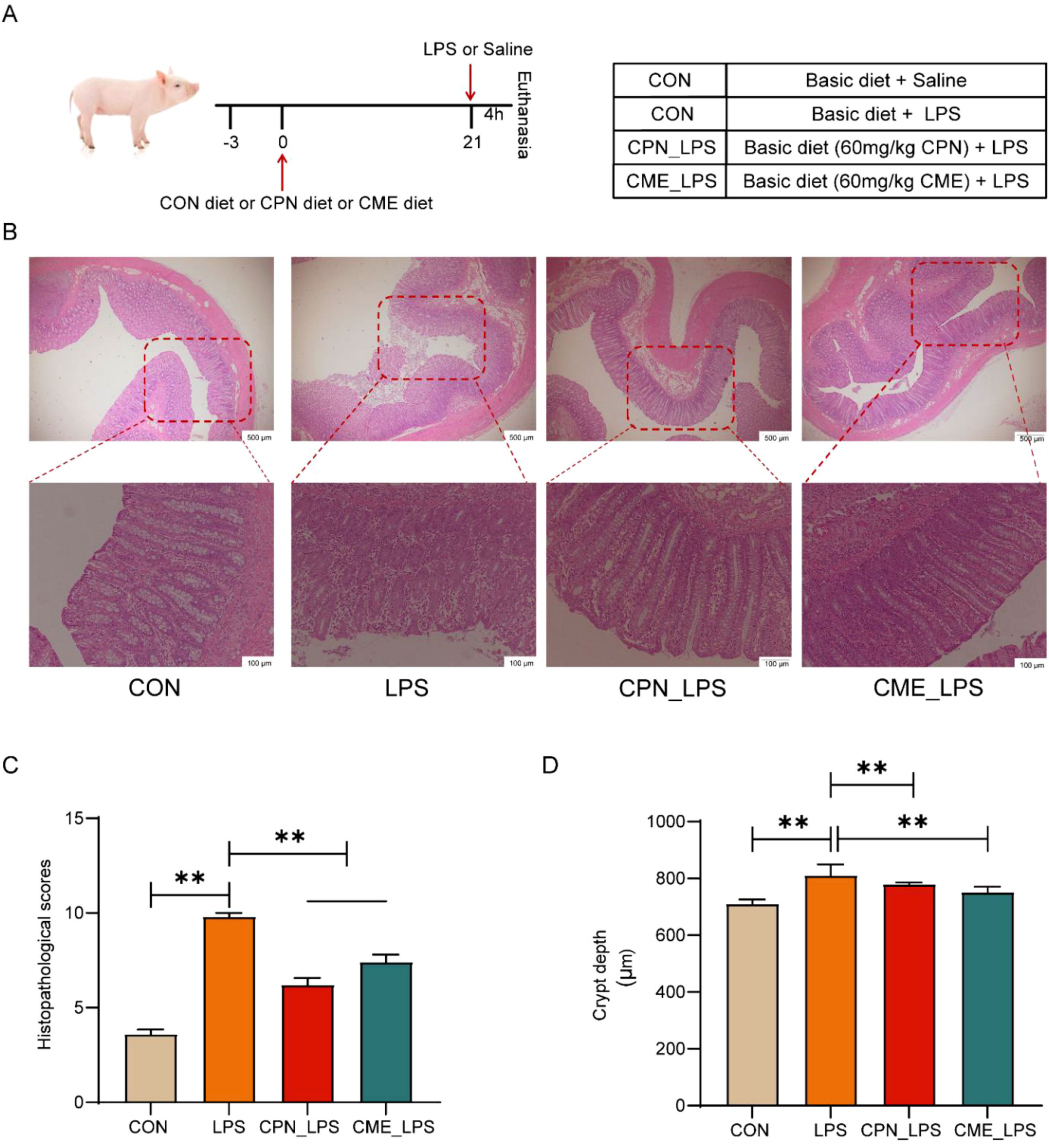
CPN/CME ameliorates LPS-challenged colonic damage in piglets. **(A)** Schematic diagram of the experimental design. **(B)** H&E staining of the colon sections (scale bar: top, 500 μm; bottom, 100 μm). **(C)** Histopathological scores of H&E-stained colonic sections. **(D)** Colon crypt depth. Data are expressed as mean ± SEM (n = 6). Statistical analysis was performed (one-way ANOVA followed by Tukey’s multiple comparisons tests) using SPSS software. *p < 0.05 and **p < 0.01. CPN, cordycepin; CME, *Cordyceps militaris* extract; LPS, lipopolysaccharide.

The animal experiment in this study was performed according to the Animal Care and Use Committee of the Shanghai Academy of Agricultural Sciences (SAASPZ0522050). The detailed experimental procedures are depicted in **Figure 1A**. All 24 weaned castrated piglets (Duroc × Landrace × Large White) were weaned at 21 days of age and were randomly assigned to four groups with six replicates per group (n = 6): control group (basal diets), LPS group (basal diets), CPN-LPS group (basal diets + 60 mg/kg cordycepin), and CME-LPS group (basal diets + 60 mg/kg *C. militaris* extract). The piglets had a 3-day acclimatization phase followed by a 21-day experimental period. There were six individual pens per group, with each pen housing a single piglet. The fundamental diet was prepared in compliance with the NRC 2012 (26) standards to fulfill the nutritional needs of piglets, with the composition and nutrient content listed in **Supplementary Table S1**. Piglets in all groups were fed daily at 7:00, 12:00, and 17:00, with continuous access to water. On day 21, groups treated with LPS, CPN-LPS, and CME-LPS received an injection of 100 μg/kg BW LPS (*Escherichia coli* O55:B5, Sigma Chemical Inc., St. Louis, MO, USA) following established protocols, whereas the CON group was administered an identical volume of sterile saline solution (20).

### 2.3 Sample collection

All piglets were euthanized 4 h post the LPS challenge; blood samples (approximately 5 mL) from the jugular vein were collected in sterile vacuum tubes, centrifuged, and stored. For colonic samples, a 4-cm mid-colon segment was excised. Mucosa samples were collected by gently scraping the inner surface of this segment with a sterile slide, then flash-frozen in liquid nitrogen, and stored at −80°C for immunological analysis. Subsequently, colon digesta (approximately 2–3 g) from the same mid-colon segment were also collected and stored at −80°C for analysis of gut microbes, metabolomics of digests, and short-chain fatty acids (SCFAs). Approximately 4 cm segments of the mid-colon were excised and fixed in 4% paraformaldehyde for morphological analysis.

### 2.4 Histopathological analysis of the colon

Colonic specimens were immersed in 4% paraformaldehyde and fixed for 24 h. Following fixation, 4-μm paraffin sections were prepared and stained with hematoxylin and eosin (H&E), and then colonic morphology was observed under a microscope (Olympus, Tokyo, Japan) to assess colonic injury and inflammation. Crypt depth (CD) measurements were conducted using the ImageJ software (Version 1.53, National Institutes of Health, Bethesda, MD, USA) and scored using histological scoring criteria (**Supplementary Table S2**) on colon sections (27).

### 2.5 Serum biochemical indicators

Serum biochemical indicators [total protein (TP), albumin (ALB), globulin (GLB), albumin-to-globulin ratio (AGR), alkaline phosphatase (ALP), lactate dehydrogenase (LDH), total cholesterol (TC), triglyceride (TG), high-density lipoprotein cholesterol (HDL-C), and low-density lipoprotein cholesterol (LDL-C)] were assayed using AU680 Automatic Biochemistry Analyser (Beckman Coulter Inc., Brea, CA, USA).

### 2.6 Colonic cytokine levels

Interleukin-1β (IL-1β), interleukin-6 (IL-6), interleukin-8 (IL-8), interleukin-10 (IL-10), and tumor necrosis factor-α (TNF-α) in colonic mucosa samples were detected using ELISA kits (Enzyme-linked Biotechnology, Shanghai, China). All cytokine determinations were strictly conducted in accordance with the manufacturer’s instructions for each commercial kit.

### 2.7 Colonic microbiota analysis

Microbial DNA from the colonic digesta was extracted using a DNA extraction kit (Axygen Biosciences, Union City, CA, USA) in accordance with the manufacturer’s protocol. The 16S rRNA gene V4–V5 region was then amplified using specific primers (515F 5′-barcode-GTGCCAGCMGCCGCGG-3′ and 907R 5′-CCGTCAATTCMTTTRAGTTT-3′). After purification using commercial kits (Axygen Biosciences, Union City, CA, USA), the PCR amplification products were quantified, and qualified libraries were sequenced on the Illumina MiSeq platform (Illumina, San Diego, CA, USA). The UPARSE software was used to construct different operational taxonomic units (OTUs) and sequence similarity thresholds greater than 97%. Alpha-diversity indices were analyzed based on Mothur (Version 1.21.1), and beta diversity was calculated through the Bray–Curtis and R package (Version 2.0). Differences in biomarkers and microbiota abundance of colonic microbiota were evaluated by linear discriminant analysis (LDA), along with the Kruskal–Wallis test and rank tests.

### 2.8 Metabolomics analysis

The colonic content samples were delivered to Novogene (Beijing, China) to employ untargeted metabolomics (Thermo Fisher, Dreieich, Germany). A colonic tissue sample measuring 100 mg was ground into powder and resuspended in 80% ice-methanol. After centrifugation (15,000 *g*, 20 min, 4°C), the supernatant was obtained and diluted with water to acquire a final concentration of 53% methanol prior to further centrifugation. Liquid chromatography–tandem mass spectrometry (LC-MS/MS) was then employed for quantification, and the initial data acquired were processed and underwent quantitative analysis through the Compound Discoverer (CD3.3, Thermo Fisher) per metabolite. These metabolites were annotated using the Kyoto Encyclopedia of Genes and Genomes (KEGG) database (https://www.genome.jp/kegg/pathway.html). Principal component analysis (PCA) and partial least squares discriminant analysis (PLS-DA) were conducted using metaX (http://metax.genomics.cn/). Univariate analysis (t-test) was performed to calculate statistical significance (*p*-value). Metabolites with a variable importance in projection (VIP) score > 1, *p*-value < 0.05, and a fold change (FC) ≥ 2 or FC ≤ 0.5 were classified as differential metabolites. Volcano plots were generated to filter metabolites of interest based on log2(FC) and −log10(*p*-value) using ggplot2 in R language.

### 2.9 SCFAs of colonic contents

Quantification of SCFA concentrations was according to our previously described methods (20). Briefly, 0.1 g of colon digesta was dissolved, shaken (30 min), and centrifuged (12,000 *g*, 10 min). The extracted supernatant was mixed with metaphosphoric acid and crotonic acid and then stored at −20°C overnight. Prior to the assay, the mixture was allowed to equilibrate at room temperature, followed by filtration and centrifugation; 0.06 µm of the supernatant was extracted and injected into a gas chromatograph (GC) (Shimadzu, Kyoto, Japan) to analyze SCFAs.

### 2.10 Statistical analysis

All data were counted and analyzed using SPSS 20.0 (SPSS Inc., Chicago, IL, USA) employing ANOVA with Tukey’s multiple comparisons tests. *p*-Value < 0.05 (* *p* < 0.05 and ** *p* < 0.01) was deemed statistically significant, while 0.05 ≤ *p*-value < 0.10 was used to indicate a trend. Spearman’s correlation analysis was conducted using the corrplot package in the R software (Version 3.0.3). The results were considered to be significantly relevant if the *p* < 0.05, and absolute r > 0.5.

## 3 Results

### 3.1 Supplementation with CPN and CME alleviated colonic morphological damage in LPS-challenged piglets

Our previous findings demonstrated that experiment piglets remained in optimal condition throughout the rearing phase. No significant differences were measured in growth parameters and diarrhea rate among different groups before the LPS injection (20). Furthermore, histological analysis of the colonic tissue revealed more disruption of crypt structure, and extensive immune cell infiltration in the mucosa and submucosa in the LPS-challenged group affirmed the establishment of a model of LPS-challenged colonic injury (**Figure 1B**). Conversely, compared to the LPS group, CPN and CME mitigated LPS-challenged crypt structure damage and inflammatory cell infiltration in piglets (**Figure 1B**). Consistent with these observations, the LPS-challenged group significantly elevated overall disease severity scores and colonic crypt depth compared with the CON group.

Additionally, interventional treatments for CPN and CME inhibited the LPS-triggered increase in overall disease severity scores and colonic crypt depth (*p* < 0.05; **Figures 1C, D**). Taken together, the above results demonstrated that the distinct treatment groups were capable of alleviating LPS-challenged colonic inflammation and mucosal damage in piglets.

### 3.2 Supplementation with CPN and CME alleviated serum metabolic dysregulation and inflammation by LPS challenge

**Figure 2:**
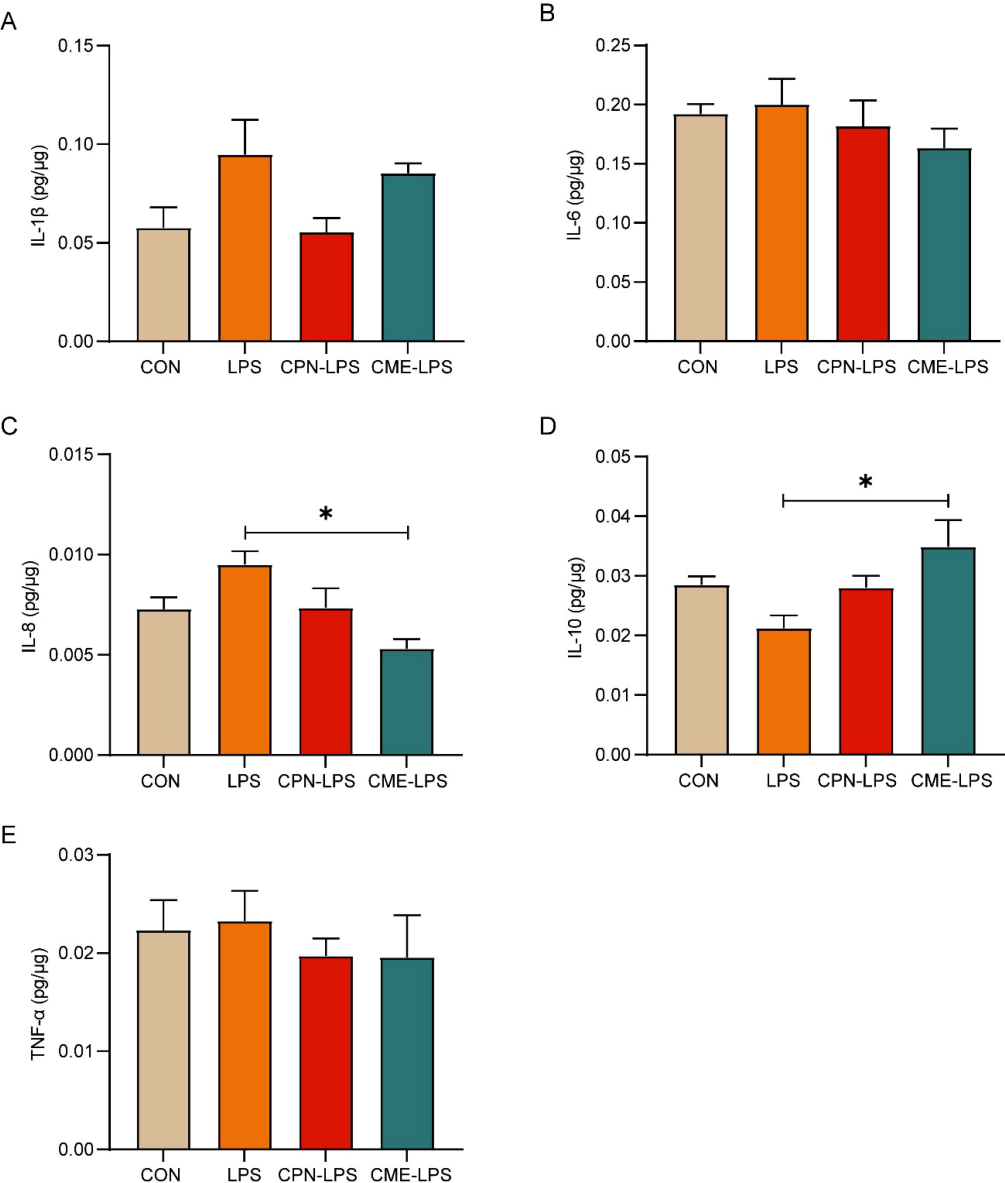
CPN/CME relieved LPS-challenged inflammatory response. **(A)** Colon IL-1β level. **(B)** Colon IL-6 level. **(C)** Colon IL-8 level. **(D)** Colon IL-10 level. **(E)** Colon TNF-α level. Data are expressed as mean ± SEM (n = 6). Statistical analysis was performed (one-way ANOVA followed by Tukey’s multiple comparisons tests) using SPSS software. *p < 0.05 and **p < 0.01. CPN, cordycepin; CME, *Cordyceps militaris* extract; LPS, lipopolysaccharide.

Intraperitoneal injection of LPS induces serum metabolic disorder and intestinal inflammatory response, ultimately leading to a disruption of the organism’s immune homeostasis (28, 29). In this experiment, we measured the changes in serum biochemical parameters and colonic mucosal cytokine levels to appraise the implications of CPN or CME on the inflammatory response by LPS stimulation. As shown in **Table 1**, the TP and GLB contents in the serum of piglets subjected to LPS-induced injury were plainly decreased in contrast to those in the CON group (*p* < 0.05). Furthermore, there was a notable elevation in AGR (*p* < 0.05), and TG concentration expressed a relatively increasing trend in LPS-challenged piglets (*p* = 0.09). Simultaneously, in comparison to the control group, the serum LDL-C content of piglets in both the LPS and CME-LPS groups exhibited a downward trend (*p* = 0.08). In sum, both of these interventions demonstrated efficacy in constraining serum metabolic disturbances in subjects with the LPS challenge. Notably, the CME treatment significantly reduced TC concentration in comparison to the LPS group (*p* < 0.05). The assessment of inflammatory cytokine levels in the colon was conducted following the administration of the LPS challenge (**Figure 2**). The result exposed that IL-8 content was remarkably improved and the levels of IL-10 markedly declined in the LPS group compared to the CME-LPS group (*p* < 0.05; **Figures 2C, D**). Moreover, the LPS challenge led to increased levels of cellular inflammatory factors (IL-1β, IL-6, and TNF-α), although these differences did not reach statistical significance (*p* > 0.05; **Figures 2A, B, E**). Generally speaking, the aforementioned results showed that CPN and CME have the capacity to restore metabolic disorder and inflammatory response under the LPS challenge.

**Table 1:** Effect of CPN/CME on serum biochemical indices in LPS-challenged piglets^1^.

Item	Treatment				SEM	*p*-Value
	CON	LPS	CPN-LPS	CME-LPS		
TP, g/L	35.30^b^	28.00^a^	32.27^ab^	37.45^b^	1.09	0.02
ALB, g/L	19.99	22.12	24.31	23.80	0.89	0.33
GLB, g/L	15.34^b^	5.88^a^	7.96^a^	13.65^b^	0.96	<0.01
AGR	1.35^a^	4.31^b^	3.55^ab^	1.76^ab^	0.35	0.04
ALP, U/L	312.25	384.70	416.00	370.92	16.90	0.14
LDH, U/L	696.20	679.47	760.68	771.77	29.67	0.72
TC, mmol/L	1.60^ab^	2.01 ^b^	1.74 ^ab^	1.46 ^a^	0.7	0.01
TG, mmol/L	0.46	0.80	0.76	0.70	0.05	0.09
HDL-C, mmol/L	0.72	0.68	0.72	0.61	0.02	0.27
LDL-C, mmol/L	1.00	0.77	0.86	0.76	0.04	0.08

CON, control; LPS, lipopolysaccharide; CPN, cordycepin; CME, *Cordyceps militaris* extract; TP, total protein; ALB, albumin; GLB, globulin; AGR, albumin-to-globulin ratio; ALP, alkaline phosphatase; LDH, lactate dehydrogenase; TC, total cholesterol; TG, triglyceride; HDL-C, high-density lipoprotein cholesterol; LDL-C, low-density lipoprotein cholesterol.

^1^Data were expressed as the mean and total SEM (n = 6). Different letters represent significant differences (*p* < 0.05) using ANOVA with Tukey’s multiple comparisons tests.

### 3.3 Alterations in colonic microbiota composition and SCFA concentrations by CPN and CME supplementation

**Figure 3:**
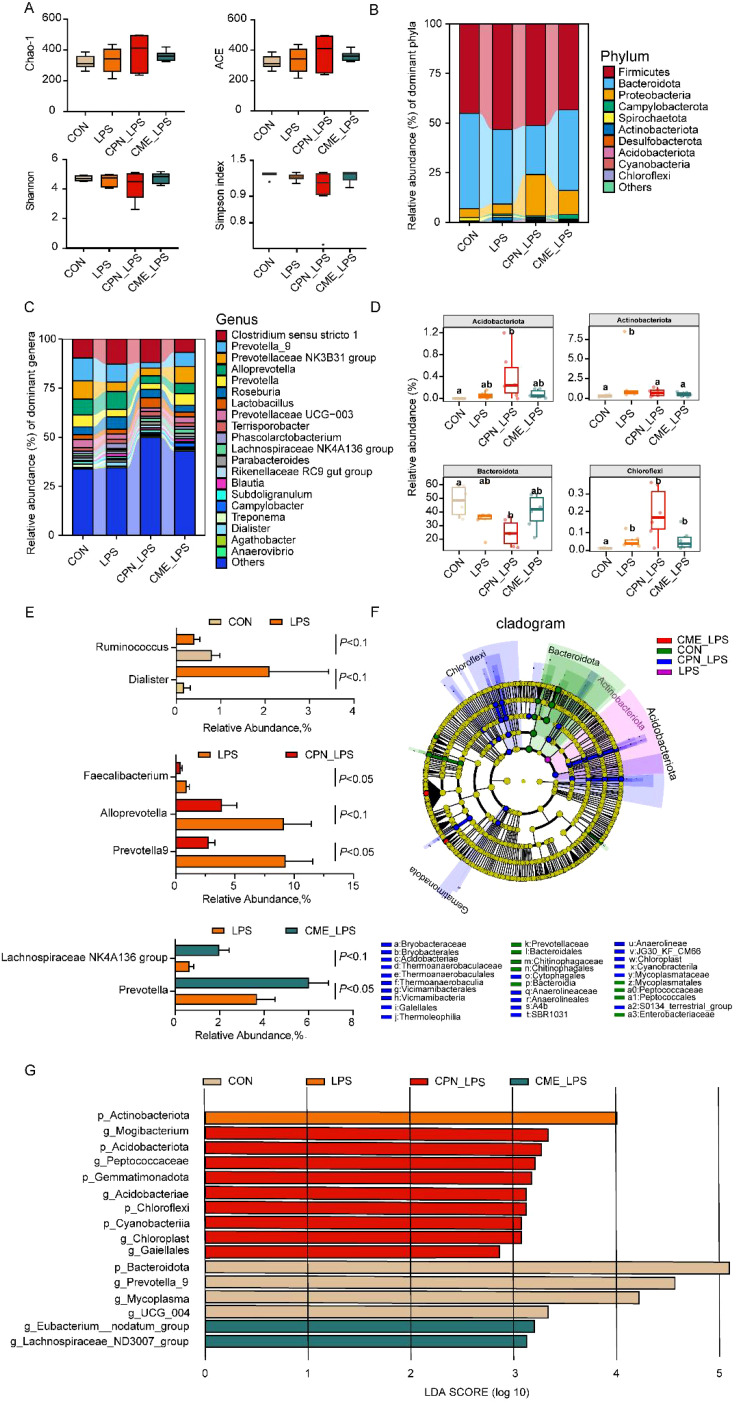
CPN/CME altered the microbial composition of the colon in LPS-challenged piglets. **(A)** Alpha diversity of colonic microbiota of different groups. Statistical analysis was performed (one-way ANOVA followed by Tukey’s multiple comparisons tests) using SPSS software. **(B, C)** The composition of colonic microbiota at phylum and genus levels. **(D)** The phylum was utilized in conducting statistical analysis on the top 10 microbiota in LPS-challenged piglets. **(E)** Colonic microorganisms with significant changes between LPS and CON groups, CPN-LPS and LPS groups, and CME-LPS and LPS groups at the genus level. Statistical analysis was performed (statistical analysis by Wilcoxon rank-sum test) using SPSS software. **(F, G)** The LEfSe analysis and LDA score plot (LDA score ≥ 2, *p* < 0.05) used to evaluate the differentially abundant taxa of colonic microbiota. Data are expressed as mean ± SEM (n = 6). CPN, cordycepin; CME, *Cordyceps militaris* extract; LPS, lipopolysaccharide; LDA, linear discriminant analysis; LEfSe, LDA effect size.

Changes in serum biochemical indicators reflect changes in the metabolic and inflammatory state of the body, which may be closely related to changes in the intestinal microbiota. Subsequently, the differences in the composition of the colonic flora of piglets in different treatment groups were examined using the 16S rRNA gene detection technique. The Venn diagram showed that the unique OTUs for the CON group, LPS group, CPN-LPS group, and CME-LPS group are 342, 451, 685, and 533, respectively (**Supplementary Figure S2A**). Principal coordinates analysis (PCoA) plots demonstrated notable clustering and differential alterations in the colonic microbiota of each group following distinct treatments (**Supplementary Figure S2B**). The alpha-diversity indices demonstrated that there was no statistically significant difference between the richness and diversity indices of the microflora among groups (*p* > 0.05; **Figure 3A**). The relative abundance of the top 10 phyla and top 20 genera is illustrated in **Figures 3B, C**. The results at the phylum level demonstrated that the Firmicutes, Bacteroidota, and Proteobacteria were preponderant, covering more than 90% of the bacterial species. Specifically, the relative abundance of Actinobacteriota in the LPS group was obviously elevated compared to that in the CON group (*P* < 0.05; **Figure 3D**). As compared with the CON group, the relative abundance of Acidobacteriota and Chloroflexi increased (*p* < 0.05), while a reduction in the relative abundance of Bacteroidota was detected in the CPN-LPS group (*p* < 0.05; **Figure 3F**). At the genus level, microbial thermogram analysis provides insight into the microbial composition of the various groups (**Supplementary Figure S1**). Specifically, the LPS challenge led to a trend of increase in the relative abundance of *Dialister* (*p* = 0.07) and a diminishing trend in the relative abundance of *Ruminococcus* (*p* = 0.09) compared with the CON group (**Figure 3E**). The abundance of *Prevotella-9* (*p* < 0.05), *Alloprevotella* (*p* = 0.07), and *Faecalibacterium* (*p* < 0.05) was lower in the CME-LPS group compared to the LPS group (**Figure 3E**). Meanwhile, the abundance of *Prevotella* (*p* = 0.09) and the *Lachnospiraceae NK4A136 group* (*p* < 0.05) was higher in the CME-LPS group compared to the LPS group (**Figure 3E**).

Biomarker taxa at different levels of colon microbiology were screened using linear discriminant analysis (LDA) effect size (LEfSe) and LDA scores across groups (**Figures 3F, G**). Bacteroidota, *UCG_004*, *Mycoplasma*, and *Prevotella-9* were enriched in the CON group; Actinobacteriota exhibited a higher representation in the LPS group; Cyanobacteria, Chloroflexi, Acidobacteriota, Gemmatimonadota, Acidobacteriae, Peptococcaceae, and *Mogibacterium* were found to be more prevalent in the CPN-LPS group; the *Lachnospiraceae ND3007 group* and *Eubacterium nodatum group* were enriched in the CPN-LPS group.

As illustrated in **Table 2**, compared to those in the CON group, the levels of both isobutyrate and isovalerate were found to markedly decrease in the LPS stress piglets (*p* < 0.05). However, CPN and CME were observed to mitigate the decline in isovalerate concentrations. No statistically obvious differences were observed in the remaining SCFA contents between the groups (*p* > 0.05).

**Table 2:** SCFA concentrations in colonic digesta^1^.

Item	Treatment				SEM	*p*-value
	CON	LPS	CPN-LPS	CME-LPS		
Acetate, μmol/g	85.70	85.99	78.57	90.52	3.92	0.73
Propionate, μmol/g	26.68	22.18	22.22	22.90	1.47	0.72
Isobutyrate, μmol/g	7.27^b^	1.09^a^	1.22^a^	2.10^a^	0.78	<0.01
Butyrate, μmol/g	14.22	13.32	15.98	14.53	1.29	0.90
Isovalerate, μmol/g	3.21^b^	1.56^a^	1.85^ab^	2.07^ab^	0.21	0.02
Valerate, μmol/g	2.94	2.2	2.83	3.03	0.27	0.79
Total SCFAs, μmol/g	140.02	126.41	122.66	135.15	6.02	0.76

SCFA, short-chain fatty acid; CON, control; LPS, lipopolysaccharide; CPN, cordycepin; CME, *Cordyceps militaris* extract.

^1^Data were expressed as the mean and total SEM (n = 6). Different letters represent significant differences (*p* < 0.05) using ANOVA with Tukey’s multiple comparisons tests.

### 3.4 Colonic digesta metabolomics

**Figure 4:**
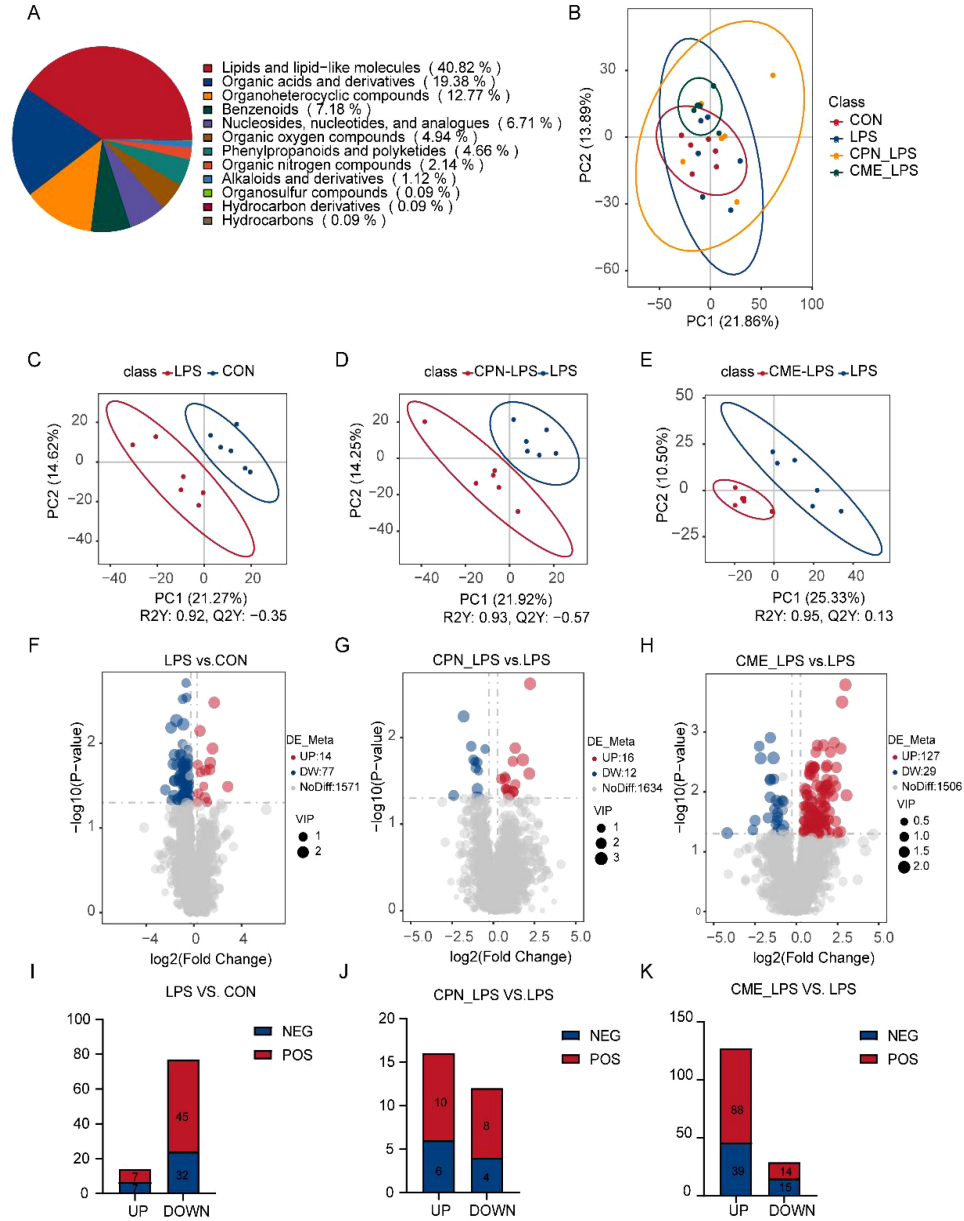
CPN/CME treatment modulates microbial metabolites in LPS-challenged piglets. **(A)** Pie chart showing the percentage of each type of microbial metabolite. **(B)** Metabolomics detection of PCoA. **(C–E)** PLS-DA score plots of LPS vs. CON, CPN-LPS vs. LPS, and CME-LPS vs. LPS. **(F–H)** The volcano plots showing the differential variables between the groups, with red dots for upregulated metabolites, blue dots for downregulated metabolites, and gray dots for metabolites that are not significantly different. **(I–K)** The number of differentially expressed metabolites with functional annotations (*p* < 0.05, VIP > 1). Data are expressed as mean ± SEM (n = 6). CPN, cordycepin; CME, *Cordyceps militaris* extract; LPS, lipopolysaccharide; PCoA, principal coordinates analysis; PLS-DA, partial least squares discriminant analysis; VIP, variable importance in projection.

Non-targeted metabolomics analysis was employed to elucidate the differences in colonic metabolites in piglets following LPS administration. Altogether 1,662 metabolites were identified in the course of this experiment. The metabolites were classified into the following categories: lipids and lipid-like molecules (40.82%), organic acids and derivatives (19.38%), organic heterocyclic compounds (12.77%), and benzenoids (7.18%) (**Figure 4A**). PCA score plots demonstrated notable clustering and separation within each group, indicating that the metabolites in the colon of the LPS-challenged piglets were significantly altered (**Figure 4B**). The results of the permutation test of PLS-DAs demonstrated the reliability of the groups within the model. For the comparisons between the LPS and CON groups, R^2^ = 0.92 and Q^2^ = −0.35; between the CPN-LPS and LPS groups, R^2^ = 0.93 and Q^2^ = −0.57; and between the CME-LPS and LPS groups, R^2^ = 0.95 and Q^2^ = 0.13. Significant differences were observed (**Figures 4C–E**). A screening process was conducted to identify potential colonic differential metabolites, employing a PLS-DA and a t-test. Notably, the metabolomics analysis indicated that 91 metabolites were altered in the colons of piglets in the LPS group compared to the control group. Compared to the LPS group, 28 metabolites and 156 metabolites were remarkably changed in the CPN-LPS and CME-LPS groups, respectively (**Figures 4F–H**). Within each group, the number of positive ions and negative ion metabolites with significant differences were determined separately (**Figures 4I–K**). Meanwhile, a total of 314 differentially expressed metabolites were detected in the four treatment groups and exhibited in the cluster heatmap (**Supplementary Figure S3**).

**Figure 5:**
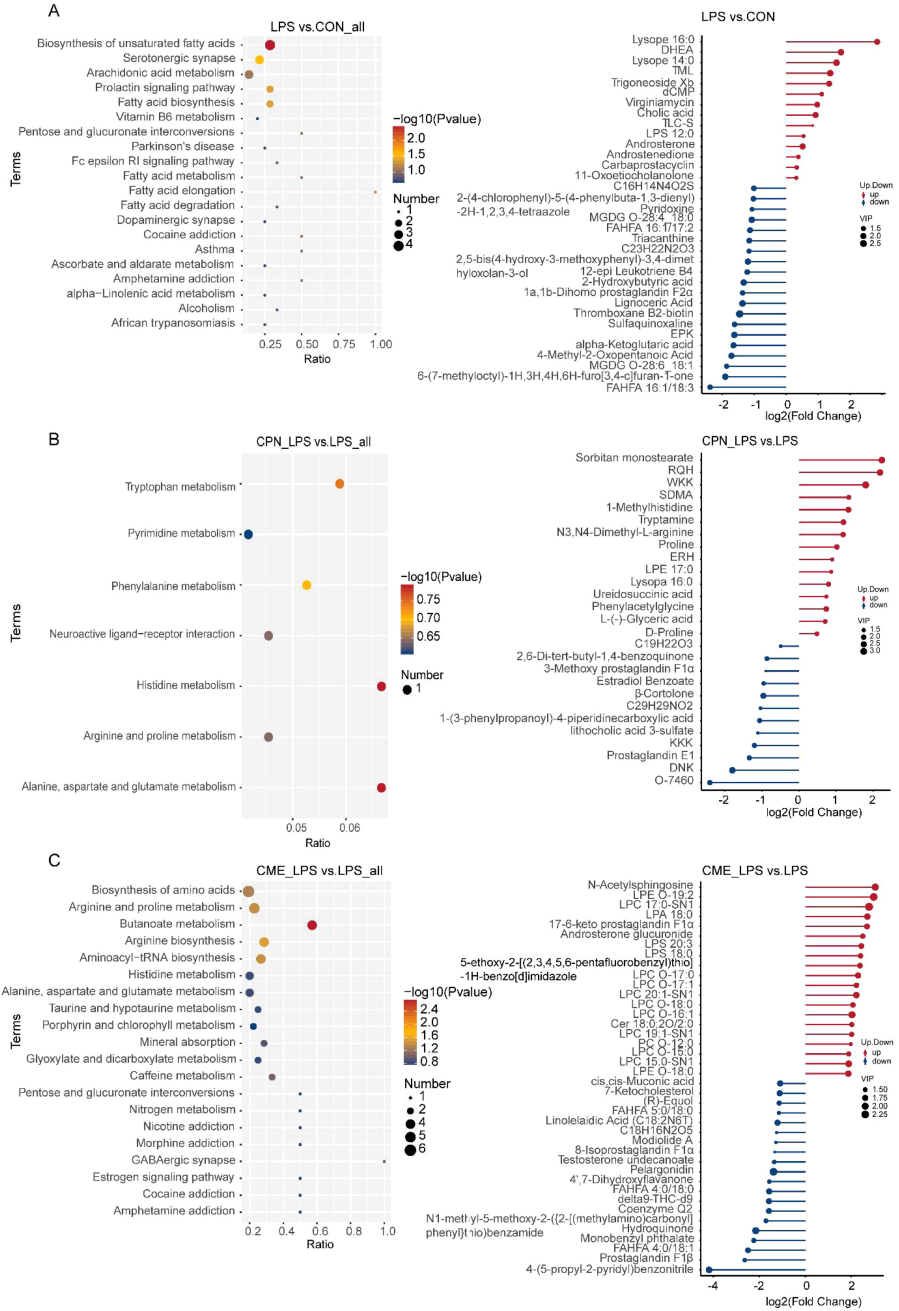
Effect of dietary CPN/CME supplementation on the differential metabolites and KEGG pathway enrichment in the colon digesta of LPS-challenged piglets. **(A)** KEGG pathway and major differential metabolites enriched between LPS and CON groups. **(B)** KEGG pathway and major differential metabolites enriched between CPN-LPS and LPS groups. **(C)** KEGG pathway and major differential metabolites enriched between CME-LPS and LPS groups. Differential upregulated metabolites and differential downregulated metabolites between the two groups will only show the top 20, and those beyond 20 will show all differential metabolites. CPN, cordycepin; CME, *Cordyceps militaris* extract; KEGG, Kyoto Encyclopedia of Genes and Genomes; LPS, lipopolysaccharide.

Finally, the enrichment of differentially expressed metabolite pathways between each two groups was analyzed using KEGG. The results demonstrated that differentially expressed metabolites were predominantly related to processes pertaining to lipid metabolism, amino acid metabolism, and bile acid metabolism (**Figures 5A–C**). The differential metabolites of palmitic acid, stearic acid, alpha-ketoglutaric acid, prostaglandin J2, prostaglandin D2, prostaglandin B2, and levodopa were inhibited, and the concentration of androstenedione significantly increased in LPS-challenged piglet colon compared with the CON group (**Figure 5A**). Furthermore, the pathways remarkably rich in differential metabolites in the CPN-LPS group were related to amino acid metabolism (including tryptophan metabolism, phenylalanine metabolism, histidine metabolism, arginine and proline metabolism, and alanine, aspartate and glutamate metabolism) when compared to the LPS group (**Figure 5B**). In CPN-LPS group piglets, metabolic pathways such as tryptamine, ureidosuccinic acid, 1-methylhistidine, phenylacetylglycine, and d-proline were enriched. Furthermore, the differential metabolites including l-glutamate, l-ornithine, 4-aminobutyric acid, *O*-phospho-l-serine, l-threonine, alpha-ketoglutaric acid, and citrulline were upregulated in the CME-LPS group, which mainly participated in biosynthesis of amino acids, arginine and proline metabolism, butanoate metabolism, arginine biosynthesis, and aminoacyl-tRNA biosynthesis (**Figure 5C**). Taken together, the LPS challenge caused intestinal lipid metabolism and amino acid metabolism disorder and led to colonic immune damage. There are differences in the modification of colonic metabolites by CPN and CME treatments, and there is a certain therapeutic effect on colonic injury caused by the LPS challenge by regulating different amino acid metabolism pathways.

### 3.5 Correlation analysis between colonic metabolites and colonic microbiota

**Figure 6:**
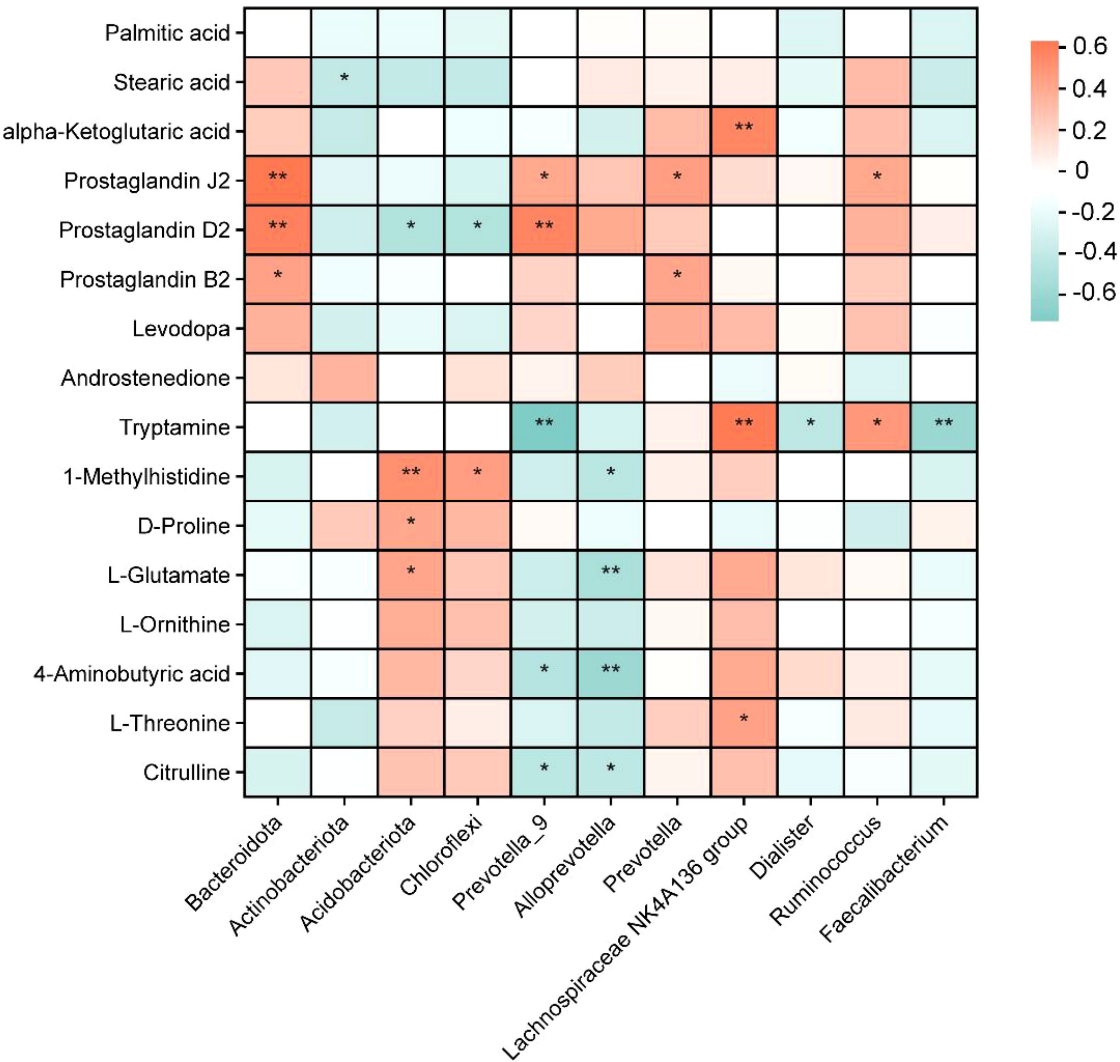
Heatmap of Spearman’s correlation analysis of colonic metabolites and colonic microbiota. Different colors represent the value of the correlation coefficient; red indicates a positive correlation, and blue indicates a negative correlation. *p < 0.05, **p < 0.01.

The potential associations between colonic metabolites and colonic microbiota were investigated by Spearman’s correlation analysis (**Figure 6**). Among the differentially abundant metabolites, Acidobacteriota and 1-methylhistidine, phenylacetylglycine, and l-glutamate were significantly positively correlated (*p* < 0.05). Tryptamine was adversely correlated with *Prevotella-9* and *Faecalibacterium* (*p* < 0.05) but proactively correlated with abundance levels in the *Lachnospiraceae ND3007 group* (*p* < 0.05). In addition, the *Lachnospiraceae ND3007 group* exhibited a negative correlation with alpha-ketoglutaric acid (*p* < 0.05).

## 4 Discussion

During the weaning period, piglets are particularly vulnerable to the immaturity of their intestinal development and the abrupt alterations in their nutritional and environmental conditions, leading to intestinal flora dysbiosis and immune regulation disorders (30). Natural bioactive substances supplements have demonstrated effectiveness as alternatives to antibiotics for improving gut health and alleviating weaning stress in pigs (31). In this study, we established an LPS-challenged intestinal injury model in piglets to investigate the effects of supplementation with CPN or CME on colonic morphology, inflammatory indices, microbiota composition, and colonic metabolomics in weaned piglets. Our findings proved that compared to those in the CON group, piglets in the LPS-challenged group exhibited structural damage to colonic crypts, significant inflammatory cell infiltration, and a notable increase in the overall disease severity score of colonic tissues. In contrast, both CPN and CME significantly improved colonic pathological symptoms and mitigated LPS-induced inflammation, in agreement with previous studies (32). Overall, these results indicate that both CPN and CME effectively facilitate the repair of intestinal damage induced by LPS in piglets.

Lipopolysaccharide has the capacity to induce disturbances in serum lipid metabolism and ultimately lead to systemic inflammatory reactions in piglets (29, 33). The present results indicated that the contents of TP and GLB were dramatically reduced, whereas there was a discernible elevation in the concentrations of TC and TG in LPS-challenged piglets. However, CPN treatment failed to restore the LPS-challenged reduction in serum GLB concentration. A previous study has indicated that LPS induction reduces total protein levels in the serum of rats (34). This reduction in serum total protein levels is associated with a robust innate immune response in the organism (35). GLB, also referred to as immunoglobulin, elicits anti-inflammatory and protective actions in response to various stimuli (36). A reduction in serum GLB levels has been demonstrated to compromise animal immunity (37). This may suggest that CME is more effective in mitigating systemic acute injury compared to CPN. The AGR is a well-established biomarker of inflammation (38). The present study demonstrated that CME significantly reduces the LPS-challenged increase in TC concentration. There are reports indicating that mice exposed to microplastics show elevated levels of TG and Total Cholesterol (TCH) in serum and liver tissues, which has been associated with the induction of hepatic dyslipidemia (39). Overall, our findings confirmed that CME or CPN supplementation confers a protective function by alleviating serum lipid metabolism disorders.

LPS stimulates the immune system, triggering an inflammatory response that alters the balance between pro-inflammatory cytokines and anti-inflammatory factors in the gut, thereby exacerbating intestinal inflammation (40). Our results demonstrated a significant increase in IL-8 levels and a notable decrease in IL-10 levels in the LPS group compared to the CME-LPS group. Concurrently, LPS induction resulted in elevated levels of IL-1, IL-6, and TNF-α, although these changes did not reach statistical significance. Findings from previous investigations have shown that the intestinal mucosal barrier was disrupted in rats with experimental colitis when inflammatory factors (such as IL-6, IL-8, IL-1β, and TNF-α) were elevated, leading to intestinal barrier dysfunction (41). IL-10 serves as a broad inhibitory cytokine, functioning to suppress the pro-inflammatory responses from both innate and adaptive immunity while preventing pathological changes associated with exacerbated immune responses (42). Collectively, these findings suggest that dietary intervention with CPN or CME suppresses the overproduction of inflammatory cytokines and thereby alleviates intestinal mucosal injury.

As an essential “organ”, the gut microbiota and its metabolites, with their specific density and abundance, are necessary to maintain immune homeostasis (43). A previous study found that *C. militaris* modifies the composition of colonic microbiota in a pig model (18). Previous studies have proved that CPN/CME can change the composition of ileum bacteria and the production of SCFAs, which could potentially contribute to inhibiting LPS-induced intestinal injury (20). In this study, two distinct interventions influenced the constitution and structure of the colonic microbiota. A study revealed that piglets with Bisphenol A (BPA)-induced intestinal inflammation exhibited elevated levels of ethanolamine, which facilitated the proliferation of various *Actinobacillus* strains and pathogenic variants, ultimately leading to an increase in *Actinobacillus* in the BPA group (44). This is analogous to the increase in the relative abundance of Actinobacteriota observed in the LPS-challenged piglet colon. The increased relative abundance of Acidobacteriota and Chloroflexi in the CPN group piglets may be beneficial in suppressing intestinal oxidative stress. Studies have pointed out that a bacterial genus widely enriched in the intestines of healthy individuals—Acidobacteriota—can inhibit oxidative stress in the intestinal tract and be involved in the body’s intestinal anti-inflammatory and antioxidant responses (45, 46). The effect of Chloroflexi in the mammalian gut has rarely been reported, but the increase in Chloroflexi abundance following melatonin treatment could indicate a potential role in shaping the gut environment or metabolite profiles (47). Clinical studies have shown a notable increase in the relative abundance of Bacteroidetes in LPS-challenged inflammation model rats, with a decline in the relative abundance of the Bacteroidetes following aspirin eugenol ester treatment, which is the same as the changes in colonic Bacteroidetes after CPN treatment (48). At the genus level, we found that LPS-induced injury enhanced the relative abundance of *Dialister*, and the relative abundance of *Ruminococcus* was decreased compared with the CON group. In the current study, *Dialister* is considered to be an intestinal pathogen, and it was found to be higher in the colon cancer group (49). *Ruminococcus* was capable of increasing the short-chain fatty acid production and G protein-coupled receptor (GPCR) expression (50). Meanwhile, CPN supplementation suppressed the relative abundance of harmful bacteria (*Prevotella-9* and *Alloprevotella*), which is consistent with previous studies showing that these bacteria are significantly enriched in DSS-induced ulcerative colitis (51, 52). The increased relative abundance of *Faecalibacterium* may be closely associated with cystic fibrosis of the colon in children (53). *Prevotella* and the *Lachnospiraceae NK4A136 group* have been demonstrated to promote intestinal mucosal repair and alleviate mouse DSS colitis by promoting SCFA contents (54). The above findings indicated that CPN and CME have the potential to impede the disruption of gut microflora induced by LPS and facilitate the colonization of beneficial bacteria to a certain extent.

LEfSe analysis further revealed that the CME-LPS group was significantly enriched in Acidobacteriae, Peptococcaceae, *Mogibacterium*, and the *Lachnospiraceae ND3007 group*, and the CPN-LPS group was enriched in the *Eubacterium nodatum group*. The relative abundance of Peptococcaceae and *Mogibacterium* was found to be actively related to the production of SCFAs, which play a pivotal character in numerous body regulatory processes, including nutrient metabolism and intestinal immunity (55, 56). A study of xylo-oligosaccharide for the treatment of ulcerative colitis demonstrated that healthy subjects exhibited a higher relative abundance of the *Lachnospiraceae ND3007 group*. It was found that the reduction of this group may inhibit the production of SCFAs (57). In the present study, a significant inverse correlation was observed between the *Eubacterium nodatum group* and the content of IL-8 in the CPN group. These findings align with those of previous studies, providing evidence that this bacterial genus probably possesses anti-inflammatory properties (58). It has been demonstrated that isobutyrate and isovalerate facilitate mucosal healing and reinforce intestinal barrier integrity in inflamed colons (59), and supplementation with CPN and CME replenished diminished isovaleric acid levels in the LPS group of piglets. This further suggested that CPN and CME possessed a specific capacity to mitigate the deleterious effects of LPS on mucosal inflammation in the gut microbiota.

The alterations in gut microbiota composition are likely to have profound impacts on the metabolic pathways in the colon, as these microorganisms are closely involved in various metabolic processes. To further understand the potential mechanisms by which CME and CPN mitigate colonic injury, we performed colonic metabolomics. A differential metabolite analysis of the colonic digestate of piglets subjected to varying treatments revealed that the metabolites were predominantly involved in lipid metabolism, amino acid metabolism, and bile acid metabolism, which were involved in host immunomodulation (29, 60). The lipid metabolism pathway (biosynthesis of unsaturated fatty acids, fatty acid biosynthesis, and butanoate metabolism) and amino acid metabolism pathway (arginine biosynthesis and alanine, aspartate and glutamate metabolism) were markedly suppressed in the LPS group. Supplementation with CPN and CME alleviated LPS-challenged blockage of amino acid metabolic pathways. Oleic acid (OA) and palmitic acid (PA) produced by *Bacteroides thetaiotaomicron* and *Lactobacillus johnsonii* reduced the content of inflammatory mediators in DSS-stimulated intestinal epithelial Caco-2 cells (61). As an important mediator of the tricarboxylic acid cycle, alpha-ketoglutaric acid plays a pivotal role in the alleviation of oxidative stress (62). Intestinal manipulation-induced inflammation in mice has been observed to result in a reduction in the expression of mRNA for prostaglandin J2 and prostaglandin D2, thereby increasing intestinal epithelial barrier permeability (63). In the present experiments, the inhibition of these metabolites in the LPS group indicated a lower reduction of anti-inflammatory capacity. As demonstrated in prior research, 1-methylhistidine exerts antioxidant properties in muscles and the brain (64), Phenylacetylglycine is the byproduct of phenylalanine metabolism and was found to impede the development of colorectal cancer (65). These metabolites were significantly affected by CPN treatment, suggesting that CPN may alleviate LPS-challenged inflammation by enhancing antioxidant capacity as well as maintaining amino acid metabolic balance. The levels of l-glutamate, l-ornithine, l-threonine, alpha-ketoglutaric acid, and citrulline were upregulated in the CME-LPS group. CME supplementation-induced upregulation of amino acid levels significantly alleviated LPS-induced colonic metabolic disturbances, which was consistent with previous studies (66–68).

Spearman’s correlation heatmap showed that Acidobacteriota and 1-methylhistidine, phenylacetylglycine, and l-glutamate were significantly positively correlated. The *Lachnospiraceae ND3007 group* was negatively correlated with alpha-ketoglutaric acid, which has been demonstrated to induce adaptive alterations in intestinal morphology in patients diagnosed with short bowel syndrome (69). l-Glutamate has been widely demonstrated to maintain mucosal barrier function in animals (70). The CME-LPS group was beneficial in maintaining intestinal structure and function by increasing the levels of l-glutamate and alpha-ketoglutaric acid. Tryptamine was negatively correlated with *Prevotella-9* and *Faecalibacterium*. Colonic microorganisms break it down into indole and indole derivatives, which help maintain blood glucose stability and lipid metabolism cycles in the body (71, 72). In the current experiments, CPN was added to increase tryptophan content and inhibit the LPS-induced elevation of the relative abundance of *Prevotella-9* and *Faecalibacterium*. This suggests that the tryptophan metabolic pathway may be involved in CPN’s mitigation.

## 5 Conclusion

In summary, CPN and CME repaired the pathological damage of the colon caused by the LPS challenge inhibiting the inflammatory response and serum metabolic disorders. At the same time, CME or CPN has the potential to alleviate colon injury by its promotion of the proliferation of acid-producing bacteria and the regulation of the metabolic pathways of tryptophan metabolism, biosynthesis of amino acids, arginine and proline metabolism, and alanine, aspartate and glutamate metabolism. Consequently, CPN and CME can be considered promising natural extracts for correcting the imbalance of the intestinal microbiota and preventing colonic damage in weaned piglets.

## Data Availability

The datasets presented in this study can be found in online repositories. The names of the repository/repositories and accession number(s) can be found in the article/**Supplementary Material**.

## References

[1] Wang G Fan Y Zhang G Cai S Ma Y Yang L. Microbiota-derived indoles alleviate intestinal inflammation and modulate microbiome by microbial cross-feeding. Microbiome. (2024) 12:59. doi: 10.1186/s40168-024-01750-y 38504383 PMC10949743

[2] Wu J Deng X Sun Y Li J Dai H Qi S. Aged oolong tea alleviates dextran sulfate sodium-induced colitis in mice by modulating the gut microbiota and its metabolites. Food Chem X. (2024) 21:101102. doi: 10.1016/j.fochx.2023.101102 38268839 PMC10805651

[3] Liu X Zhang Y Li W Yin J Zhang B Wang J. Differential responses on gut microbiota and microbial metabolome of 2’-fucosyllactose and galactooligosaccharide against DSS-induced colitis. Food Res Int. (2022) 162:112072. doi: 10.1016/j.foodres.2022.112072 36461391

[4] Paudel D Nair DVT Tian S Hao F Goand UK Joseph G. Dietary fiber guar gum-induced shift in gut microbiota metabolism and intestinal immune activity enhances susceptibility to colonic inflammation. Gut Microbes. (2024) 16:2341457. doi: 10.1080/19490976.2024.2341457 38630030 PMC11028019

[5] Wang C Wei S Liu B Wang F Lu Z Jin M. Maternal consumption of a fermented diet protects offspring against intestinal inflammation by regulating the gut microbiota. Gut Microbes. (2022) 14:2057779. doi: 10.1080/19490976.2022.2057779 35506256 PMC9090288

[6] Nylund L Satokari R Salminen S de Vos WM. Intestinal microbiota during early life - impact on health and disease. Proc Nutr Soc. (2014) 73:457–69. doi: 10.1017/S0029665114000627 24902044

[7] Zhao DD Gai YD Li C Fu ZZ Yin DQ Xie M. Dietary taurine effect on intestinal barrier function, colonic microbiota and metabolites in weanling piglets induced by LPS. Front Microbiol. (2023) 14:1259133. doi: 10.3389/fmicb.2023.1259133 38188568 PMC10770862

[8] Ruvinov I Nguyen C Scaria B Vegh C Zaitoon O Baskaran K. Lemongrass extract possesses potent anticancer activity against human colon cancers, inhibits tumorigenesis, enhances efficacy of FOLFOX, and reduces its adverse effects. Integr Cancer Ther. (2019) 18:1534735419889150. doi: 10.1177/1534735419889150 31845598 PMC6918039

[9] Zhang J Wang H Meng S Zhang C Guo L Miao Z. The effects of poria cocos polysaccharides on growth performance, immunity, and cecal microflora composition of weaned piglets. Anim (Basel). (2024) 2024:14. doi: 10.3390/ani14071121 PMC11011092 38612361

[10] Tuohy KM Probert HM Smejkal CW Gibson GR. Using probiotics and prebiotics to improve gut health. Drug Discovery Today. (2003) 8:692–700. doi: 10.1016/S1359-6446(03)02746-6 12927512

[11] Kavanova K Kostovova I Moravkova M Kubasova T Crhanova M. *In vitro* characterization of lactic acid bacteria and bifidobacteria from wild and domestic pigs: probiotic potential for post-weaning piglets. BMC Microbiol. (2025) 25:8. doi: 10.1186/s12866-024-03711-9 39789429 PMC11715547

[12] Jukic Peladic N Dell’Aquila G Carrieri B Maggio M Cherubini A Orlandoni P. Potential role of probiotics for inflammaging: A narrative review. Nutrients. (2021) 2021:13. doi: 10.3390/nu13092919 PMC8471548 34578796

[13] Zeng J Zhou Y Lyu M Huang X Xie M Huang M. Cordyceps militaris: A novel mushroom platform for metabolic engineering. Biotechnol Adv. (2024) 74:108396. doi: 10.1016/j.bioteChadv.2024.108396 38906495

[14] Liang Z Zhang K Guo H Tang X Chen M Shi J. Cordycepin alleviates hepatic fibrosis in association with the inhibition of glutaminolysis to promote hepatic stellate cell senescence. Int Immunopharmacol. (2024) 132:111981. doi: 10.1016/j.intimp.2024.111981 38565039

[15] Zhang Y Zhang G Ling J. Medicinal fungi with antiviral effect. Molecules. (2022) 27:4457–484. doi: 10.3390/molecules27144457 PMC9322162 35889330

[16] Chen L Zheng X Huang H Feng C Wu S Chen R. Cordycepin synergizes with CTLA-4 blockade to remodel the tumor microenvironment for enhanced cancer immunotherapy. Int Immunopharmacol. (2023) 124:110786. doi: 10.1016/j.intimp.2023.110786 37611443

[17] Priya PS Murugan R Almutairi BO Arokiyaraj S Shanjeev P Arockiaraj J. Delineating the protective action of cordycepin against cadmium induced oxidative stress and gut inflammation through downregulation of NF-kappaB pathway. Environ Toxicol Pharmacol. (2023) 102:104246. doi: 10.1016/j.etap.2023.104246 37595934

[18] Zheng H Cao H Zhang D Huang J Li J Wang S. Cordyceps militaris modulates intestinal barrier function and gut microbiota in a pig model. Front Microbiol. (2022) 13:810230. doi: 10.3389/fmicb.2022.810230 35369439 PMC8969440

[19] Han ES Oh JY Park HJ. Cordyceps militaris extract suppresses dextran sodium sulfate-induced acute colitis in mice and production of inflammatory mediators from macrophages and mast cells. J Ethnopharmacol. (2011) 134:703–10. doi: 10.1016/j.jep.2011.01.022 21277968

[20] Xiong S Jiang J Wan F Tan D Zheng H Xue H. Cordyceps militaris extract and cordycepin alleviate oxidative stress, modulate gut microbiota and ameliorate intestinal damage in LPS-induced piglets. Antioxid (Basel). (2024) 13:13. doi: 10.3390/antiox13040441 PMC11047340 38671889

[21] Akbari E Asemi Z Daneshvar Kakhaki R Bahmani F Kouchaki E Tamtaji OR. Effect of probiotic supplementation on cognitive function and metabolic status in Alzheimer’s disease: A randomized, Double-Blind and Controlled Trial. Front Aging Neurosci. (2016) 8:256. doi: 10.3389/fnagi.2016.00256 27891089 PMC5105117

[22] Arora K Green M Prakash S. The microbiome and Alzheimer’s disease: potential and limitations of prebiotic, synbiotic, and probiotic formulations. Front Bioeng Biotechnol. (2020) 8:537847. doi: 10.3389/fbioe.2020.537847 33384986 PMC7771210

[23] Lin A Yan X Xu R Wang H Su Y Zhu W. Effects of lactic acid bacteria-fermented formula milk supplementation on colonic microbiota and mucosal transcriptome profile of weaned piglets. Animal. (2023) 17:100959. doi: 10.1016/j.animal.2023.100959 37688970

[24] Li K Xiao Y Chen J Chen J He X Yang H. Microbial composition in different gut locations of weaning piglets receiving antibiotics. Asian-Australas J Anim Sci. (2017) 30:78–84. doi: 10.5713/ajas.16.0285 27383806 PMC5205595

[25] Liu Q He M Zeng Z Huang X Fang S Zhao Y. Extensive identification of serum metabolites related to microbes in different gut locations and evaluating their associations with porcine fatness. Microb Biotechnol. (2023) 16:1293–311. doi: 10.1111/1751-7915.14245 PMC10221527 36916818

[26] C. National Research. Nutrient Requirements of Swine: Eleventh Revised Edition. Washington, DC: The National Academies Press (2012).

[27] Zhou L Liu D Xie Y Yao X Li Y. Bifidobacterium infantis induces protective colonic PD-L1 and foxp3 regulatory T cells in an acute murine experimental model of inflammatory bowel disease. Gut Liver. (2019) 13:430–9. doi: 10.5009/gnl18316 PMC6622561 30600673

[28] Cui C Wei Y Wang Y Ma W Zheng X Wang J. Dietary supplementation of benzoic acid and essential oils combination enhances intestinal resilience against LPS stimulation in weaned piglets. J Anim Sci Biotechnol. (2024) 15:4. doi: 10.1186/s40104-023-00958-6 38238856 PMC10797991

[29] Tian S Wang J Gao R Zhao F Wang J Zhu W. Galacto-oligosaccharides alleviate LPS-induced immune imbalance in small intestine through regulating gut microbe composition and bile acid pool. J Agric Food Chem. (2023) 71:17615–26. doi: 10.1021/acs.jafc.3c00419 37947505

[30] Huangfu W Ma J Zhang Y Liu M Liu B Zhao J. Dietary fiber-derived butyrate alleviates piglet weaning stress by modulating the TLR4/myD88/NF-kappaB pathway. Nutrients. (2024) 16:1714–37. doi: 10.3390/nu16111714 PMC11174469 38892647

[31] Huang J Qin W Xu B Sun H Jing F Xu Y. Rice bran oil supplementation protects swine weanlings against diarrhea and lipopolysaccharide challenge. J Zhejiang Univ Sci B. (2023) 24:430–41. doi: 10.1631/jzus.B2200565 PMC10186138 37190892

[32] Chen J Wang M Zhang P Li H Qu K Xu R. Cordycepin alleviated metabolic inflammation in Western diet-fed mice by targeting intestinal barrier integrity and intestinal flora. Pharmacol Res. (2022) 178:106191. doi: 10.1016/j.phrs.2022.106191 35346845

[33] Gao R Tian S Wang J Zhu W. Galacto-oligosaccharides improve barrier function and relieve colonic inflammation via modulating mucosa-associated microbiota composition in lipopolysaccharides-challenged piglets. J Anim Sci Biotechnol. (2021) 12:92. doi: 10.1186/s40104-021-00612-z 34376253 PMC8356462

[34] Khodir AE Ghoneim HA Rahim MA Suddek GM. Montelukast attenuates lipopolysaccharide-induced cardiac injury in rats. Hum Exp Toxicol. (2016) 35:388–97. doi: 10.1177/0960327115591372 26089034

[35] Li MY Zhu XM Niu XT Chen XM Tian JX Kong YD. Effects of dietary Allium mongolicum Regel polysaccharide on growth, lipopolysaccharide-induced antioxidant responses and immune responses in Channa argus. Mol Biol Rep. (2019) 46:2221–30. doi: 10.1007/s11033-019-04677-y 30747383

[36] Wu X Liu Y Tu D Liu X Niu S Suo Y. Role of NLRP3-inflammasome/caspase-1/galectin-3 pathway on atrial remodeling in diabetic rabbits. J Cardiovasc Transl Res. (2020) 13:731–40. doi: 10.1007/s12265-020-09965-8 32048199

[37] Li A Wang Y Li Z Qamar H Mehmood K Zhang L. Probiotics isolated from yaks improves the growth performance, antioxidant activity, and cytokines related to immunity and inflammation in mice. Microb Cell Fact. (2019) 18:112. doi: 10.1186/s12934-019-1161-6 31217027 PMC6585042

[38] Wang H Zhou H Jiang R Qian Z Wang F Cao L. Globulin, the albumin-to-globulin ratio, and fibrinogen perform well in the diagnosis of Periprosthetic joint infection. BMC Musculoskelet Disord. (2021) 22:583. doi: 10.1186/s12891-021-04463-7 34172035 PMC8235840

[39] Zhuang J Chen Q Xu L Chen X. Combined exposure to polyvinyl chloride and polystyrene microplastics induces liver injury and perturbs gut microbial and serum metabolic homeostasis in mice. Ecotoxicol Environ Saf. (2023) 267:115637. doi: 10.1016/j.ecoenv.2023.115637 37944461

[40] Xue HH Li JJ Li SF Guo J Yan RP Chen TG. Phillygenin attenuated colon inflammation and improved intestinal mucosal barrier in DSS-induced colitis mice via TLR4/src mediated MAPK and NF-κB signaling pathways. Int J Mol Sci. (2023) 24:2238–56. doi: 10.3390/ijms24032238 PMC9917337 36768559

[41] Ji W Huo Y Zhang Y Qian X Ren Y Hu C. Palmatine inhibits expression fat mass and obesity associated protein (FTO) and exhibits a curative effect in dextran sulfate sodium (DSS)-induced experimental colitis. Int Immunopharmacol. (2024) 132:111968. doi: 10.1016/j.intimp.2024.111968 38579565

[42] Hazlett LD Jiang X McClellan SA. IL-10 function, regulation, and in bacterial keratitis. J Ocul Pharmacol Ther. (2014) 30:373–80. doi: 10.1089/jop.2014.0018 PMC4043257 24738920

[43] Ghosh S Whitley CS Haribabu B Jala VR. Regulation of intestinal barrier function by microbial metabolites. Cell Mol Gastroenterol Hepatol. (2021) 11:1463–82. doi: 10.1016/j.jcmgh.2021.02.007 PMC8025057 33610769

[44] Liu Z Liu M Wang H Qin P Di Y Jiang S. Glutamine attenuates bisphenol A-induced intestinal inflammation by regulating gut microbiota and TLR4-p38/MAPK-NF-kappaB pathway in piglets. Ecotoxicol Environ Saf. (2024) 270:115836. doi: 10.1016/j.ecoenv.2023.115836 38154151

[45] Wang W Zhang J Li Y Su S Wei L Li L. Lactoferrin alleviates chronic low−grade inflammation response in obese mice by regulating intestinal flora. Mol Med Rep. (2024) 30:138–49. doi: 10.3892/mmr.2024.13262 PMC11200051 38873986

[46] Wang W Wang Y Duan Y Meng Z An X Qi J. Regulation of wheat bran feruloyl oligosaccharides in the intestinal antioxidative capacity of rats associated with the p38/JNK-Nrf2 signaling pathway and gut microbiota. J Sci Food Agric. (2022) 102:6992–7002. doi: 10.1002/jsfa.v102.15 35689477

[47] Zheng J Zhou Y Zhang D Ma K Gong Y Luo X. Intestinal melatonin levels and gut microbiota homeostasis are independent of the pineal gland in pigs. Front Microbiol. (2024) 15:1352586. doi: 10.3389/fmicb.2024.1352586 38596375 PMC11003461

[48] Tao Q Liu XW Zhang ZD Ma N Lu XR Ge WB. Protective effect and mechanism of aspirin eugenol ester on lipopolysaccharide-induced intestinal barrier injury. Int J Mol Sci. (2023) 24:17434–64. doi: 10.3390/ijms242417434 PMC10743450 38139262

[49] Mahdy MS Azmy AF Dishisha T Mohamed WR Ahmed KA Hassan A. Irinotecan-gut microbiota interactions and the capability of probiotics to mitigate Irinotecan-associated toxicity. BMC Microbiol. (2023) 23:53. doi: 10.1186/s12866-023-02791-3 36864380 PMC9979425

[50] Deng J Zou X Liang Y Zhong J Zhou K Zhang J. Hypoglycemic effects of different molecular weight konjac glucomannans via intestinal microbiota and SCFAs mediated mechanism. Int J Biol Macromol. (2023) 234:122941. doi: 10.1016/j.ijbiomac.2022.12.160 36563827

[51] Luo Y Fu S Liu Y Kong S Liao Q Lin L. Banxia Xiexin decoction modulates gut microbiota and gut microbiota metabolism to alleviate DSS-induced ulcerative colitis. J Ethnopharmacol. (2024) 326:117990. doi: 10.1016/j.jep.2024.117990 38423412

[52] Yu S Guo H Ji Z Zheng Y Wang B Chen Q. Sea cucumber peptides ameliorate DSS-induced ulcerative colitis: the role of the gut microbiota, the intestinal barrier, and macrophage polarization. Nutrients. (2023) 15:4813–31. doi: 10.3390/nu15224813 PMC10674221 38004208

[53] Asensio-Grau A Heredia A Garcia-Hernandez J Cabrera-Rubio R Masip E Ribes-Koninckx C. Effect of beta-glucan supplementation on cystic fibrosis colonic microbiota: an *in vitro* study. Pediatr Res. (2024) 95:1519–27. doi: 10.1038/s41390-023-02944-0 38092964

[54] He P Zhang Y Chen R Tong Z Zhang M Wu H. The maca protein ameliorates DSS-induced colitis in mice by modulating the gut microbiota and production of SCFAs. Food Funct. (2023) 14:10329–46. doi: 10.1039/D3FO03654E 37955225

[55] Quan J Xu C Ruan D Ye Y Qiu Y Wu J. Composition, function, and timing: exploring the early-life gut microbiota in piglets for probiotic interventions. J Anim Sci Biotechnol. (2023) 14:143. doi: 10.1186/s40104-023-00943-z 37957747 PMC10641937

[56] Long C de Vries S Venema K. Differently pre-treated rapeseed meals affect *in vitro* swine gut microbiota composition. Front Microbiol. (2020) 11:570985. doi: 10.3389/fmicb.2020.570985 32983078 PMC7483658

[57] Li Z Li Z Zhu L Dai N Sun G Peng L. Effects of xylo-oligosaccharide on the gut microbiota of patients with ulcerative colitis in clinical remission. Front Nutr. (2021) 8:778542. doi: 10.3389/fnut.2021.778542 35028306 PMC8748261

[58] Wei Y Chang L Liu G Wang X Yang Y Hashimoto K. Long-lasting beneficial effects of maternal intake of sulforaphane glucosinolate on gut microbiota in adult offspring. J Nutr Biochem. (2022) 109:109098. doi: 10.1016/j.jnutbio.2022.109098 35788394

[59] Teng Y Li J Guo J Yan C Wang A Xia X. Alginate oligosaccharide improves 5-fluorouracil-induced intestinal mucositis by enhancing intestinal barrier and modulating intestinal levels of butyrate and isovalerate. Int J Biol Macromol. (2024) 276:133699. doi: 10.1016/j.ijbiomac.2024.133699 38972652

[60] Liu Z Wang H Han H Li N Zheng Z Liang S. The protective effect of dulcitol on lipopolysaccharide-induced intestinal injury in piglets: mechanistic insights. J Nutr Biochem. (2024) 133:109719. doi: 10.1016/j.jnutbio.2024.109719 39103108

[61] Charlet R Le Danvic C Sendid B Nagnan-Le-Meillour P Jawhara S. Oleic Acid and Palmitic Acid from Bacteroides thetaiotaomicron and Lactobacillus johnsonii Exhibit Anti-Inflammatory and Antifungal Properties. Microorganisms. (2022) 10:1803–23. doi: 10.3390/microorganisms10091803 PMC9504516 36144406

[62] Liu G Lu J Sun W Jia G Zhao H Chen X. Alpha-ketoglutaric acid attenuates oxidative stress and modulates mitochondrial dynamics and autophagy of spleen in a piglet model of lipopolysaccharide-induced sepsis. Free Radic Biol Med. (2024) 214:80–6. doi: 10.1016/j.freeradbiomed.2024.02.009 38346662

[63] Bessard A Cardaillac C Oullier T Cenac N Rolli-Derkinderen M Neunlist M. Alterations of prostanoid expression and intestinal epithelial barrier functions in ileus. J Surg Res. (2024) 296:165–73. doi: 10.1016/j.jss.2023.12.018 38277953

[64] Kohen R Yamamoto Y Cundy KC Ames BN. Antioxidant activity of carnosine, homocarnosine, and anserine present in muscle and brain. Proc Natl Acad Sci U.S.A. (1988) 85:3175–9. doi: 10.1073/pnas.85.9.3175 PMC280166 3362866

[65] Li W Zhang X Feng Y Han H Cai J Zhao H. Deciphering the metabolic profile and anti-colorectal cancer mechanism of Capilliposide A using ultra performance liquid chromatography mass spectrometry combined with non-targeted metabolomics studies. J Pharm BioMed Anal. (2023) 234:115548. doi: 10.1016/j.jpba.2023.115548 37390605

[66] Rosser EC Piper CJM Matei DE Blair PA Rendeiro AF Orford M. Microbiota-derived metabolites suppress arthritis by amplifying aryl-hydrocarbon receptor activation in regulatory B cells. Cell Metab. (2020) 31:837–851 e810. doi: 10.1016/j.cmet.2020.03.003 32213346 PMC7156916

[67] Ma Y Ding S Liu G Fang J Yan W Duraipandiyan V. Egg protein transferrin-derived peptides IRW and IQW regulate citrobacter rodentium-induced, inflammation-related microbial and metabolomic profiles. Front Microbiol. (2019) 10:643. doi: 10.3389/fmicb.2019.00643 31001226 PMC6456682

[68] Singh K Gobert AP Coburn LA Barry DP Allaman M Asim M. Dietary arginine regulates severity of experimental colitis and affects the colonic microbiome. Front Cell Infect Microbiol. (2019) 9:66. doi: 10.3389/fcimb.2019.00066 30972302 PMC6443829

[69] Grzesiak P Slupecka-Ziemilska M Wolinski J. The biological role of a-ketoglutaric acid in physiological processes and its therapeutic potential. Dev Period Med. (2016) 20:61–7. 27416627

[70] Hu X Zhen W Bai D Zhong J Zhang R Zhang H. Effects of dietary chlorogenic acid on cecal microbiota and metabolites in broilers during lipopolysaccharide-induced immune stress. Front Microbiol. (2024) 15:1347053. doi: 10.3389/fmicb.2024.1347053 38525083 PMC10957784

[71] He Z Guo J Zhang H Yu J Zhou Y Wang Y. Atractylodes macrocephala Koidz polysaccharide improves glycolipid metabolism disorders through activation of aryl hydrocarbon receptor by gut flora-produced tryptophan metabolites. Int J Biol Macromol. (2023) 253:126987. doi: 10.1016/j.ijbiomac.2023.126987 37729987

[72] Agus A Planchais J Sokol H. Gut microbiota regulation of tryptophan metabolism in health and disease. Cell Host Microbe. (2018) 23:716–24. doi: 10.1016/j.chom.2018.05.003 29902437

