# Supplementary material for: Cordyceps militaris extract and cordycepin ameliorate LPS-challenged colonic damage in piglets by modulating the microbiota and metabolite profiles
Source: Front Immunol. 2025 Mar 10;16:1530098. doi: 10.3389/fimmu.2025.1530098 (PMC11931037; doi:10.3389/fimmu.2025.1530098)
Supplement: Supplementary file 1 [file DataSheet1.docx]

**Supplementary materials**

**Table S1.** Composition and nutrient level of the basal diet (as fed basis).

| Ingredient | Percentage (%) | Calculated nutritional compositions (%) | |
| --- | --- | --- | --- |
| Corn | 70.0 | Digestive energy (MJ/kg) | 14.60 |
| Soybean meal | 18.0 | Crude protein | 16.00 |
| Wheat bran | 6.50 | Lysine | 1.23 |
| Soybean oil | 1.90 | Methionine+Cystine | 0.70 |
| Lysine | 0.69 | Threonine | 0.79 |
| Methionine | 0.24 | Tryptophan | 0.22 |
| Threonine | 0.30 |  |  |
| Tryptophan | 0.07 |  |  |
| Calcium hydrogen phosphate | 0.45 |  |  |
| Stone powder | 0.50 |  |  |
| Salt | 0.30 |  |  |
| Multivitamins^1^ | 0.03 |  |  |
| Minerals^2^ | 0.20 |  |  |
| Choline chloride (50%) | 0.12 |  |  |
| Zeolite powder | 0.60 |  |  |
| Antioxidant | 0.1 |  |  |
| Total | 100.0 |  |  |

^1^ The minerals supply per kg diet as follows: Fe 165 mg, Zn 165 mg, Cu 16.5 mg, Mn 30 mg, Co 0.15 mg, I 0.25 mg, Se 0.25 mg.

^2^ The multivitamins supply per kg diet as follows: VA 11 000 IU, VD3 1 000 IU, VE 16 IU, VK1 1mg, VB1 0.6 mg, VB2 0.6 mg, d-pantothenic acid 6 mg, nicotinic acid 10 mg, VB12 0.03 mg, folic acid 0.8 mg, VB6 1.5 mg.

**Table S2.** Histopathological score.

| Score | Degree of inflammation | Extent | Crypt damage | Percent involvement |
| --- | --- | --- | --- | --- |
| 0 | none | none | none | 1–25% |
| 1 | slight | mucosa | basal 1/3 damage | 26–50% |
| 2 | moderate | mucosa and submucosa | basal 2/3 damage | 51–75% |
| 3 | severe | transmural | only surface epithelium intact | 76–100% |
| 4 | — | — | entire crypt and epithelium lost | 1–25% |

**Fig. S1.** Microbial thermogram analysis reveals microbial composition among the different groups.

**Fig. S2.** Effect of CPN/CME on the microbiological composition of the colon of LPS-induced piglets. (A) Venn diagram of OTUs in colon chyme. (B) PCoA plot of colon microbiota.

**Fig. S3.** Hierarchical cluster thermal analysis of different metabolites.

**
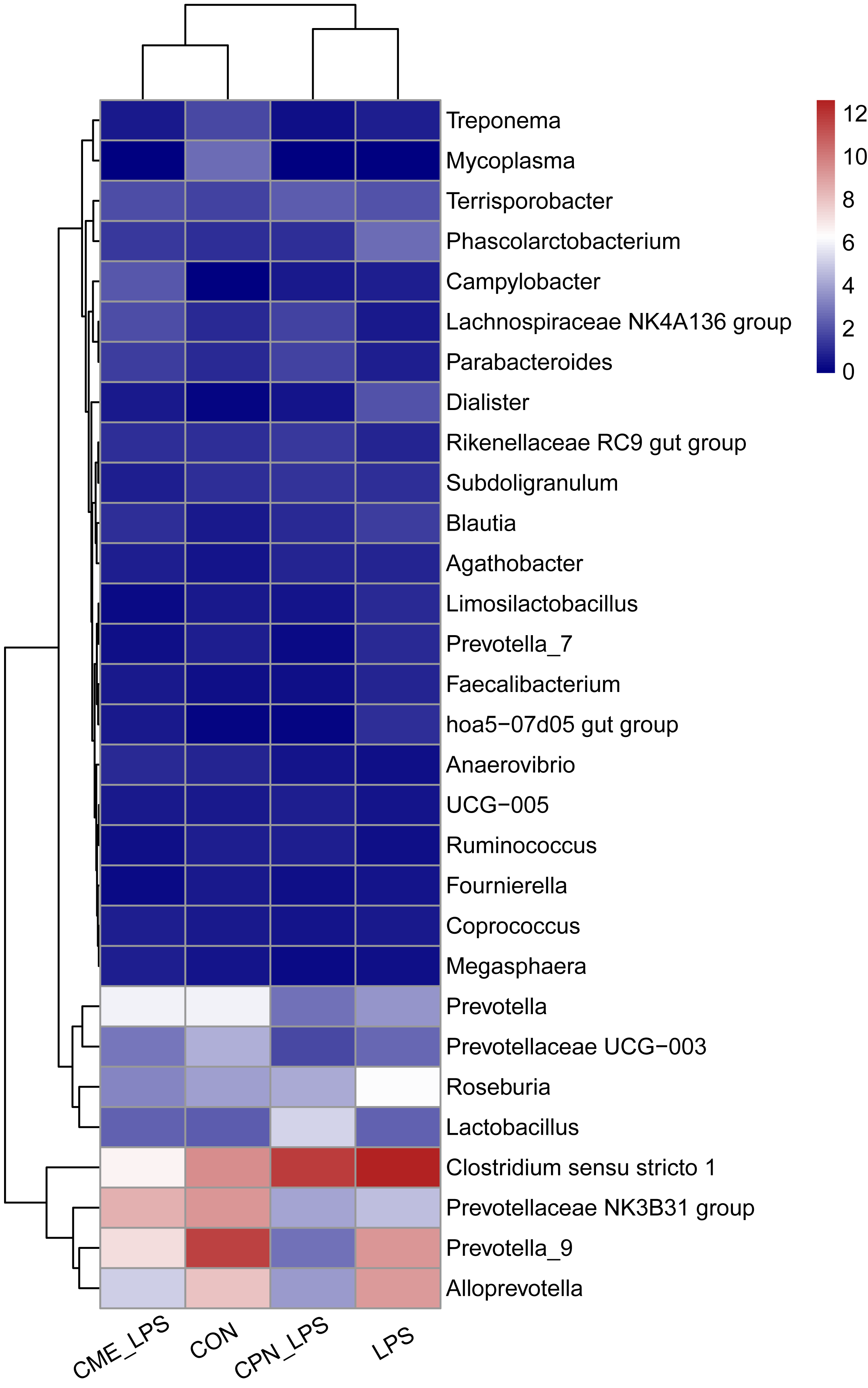
Fig.S1**

**Fig. S2**


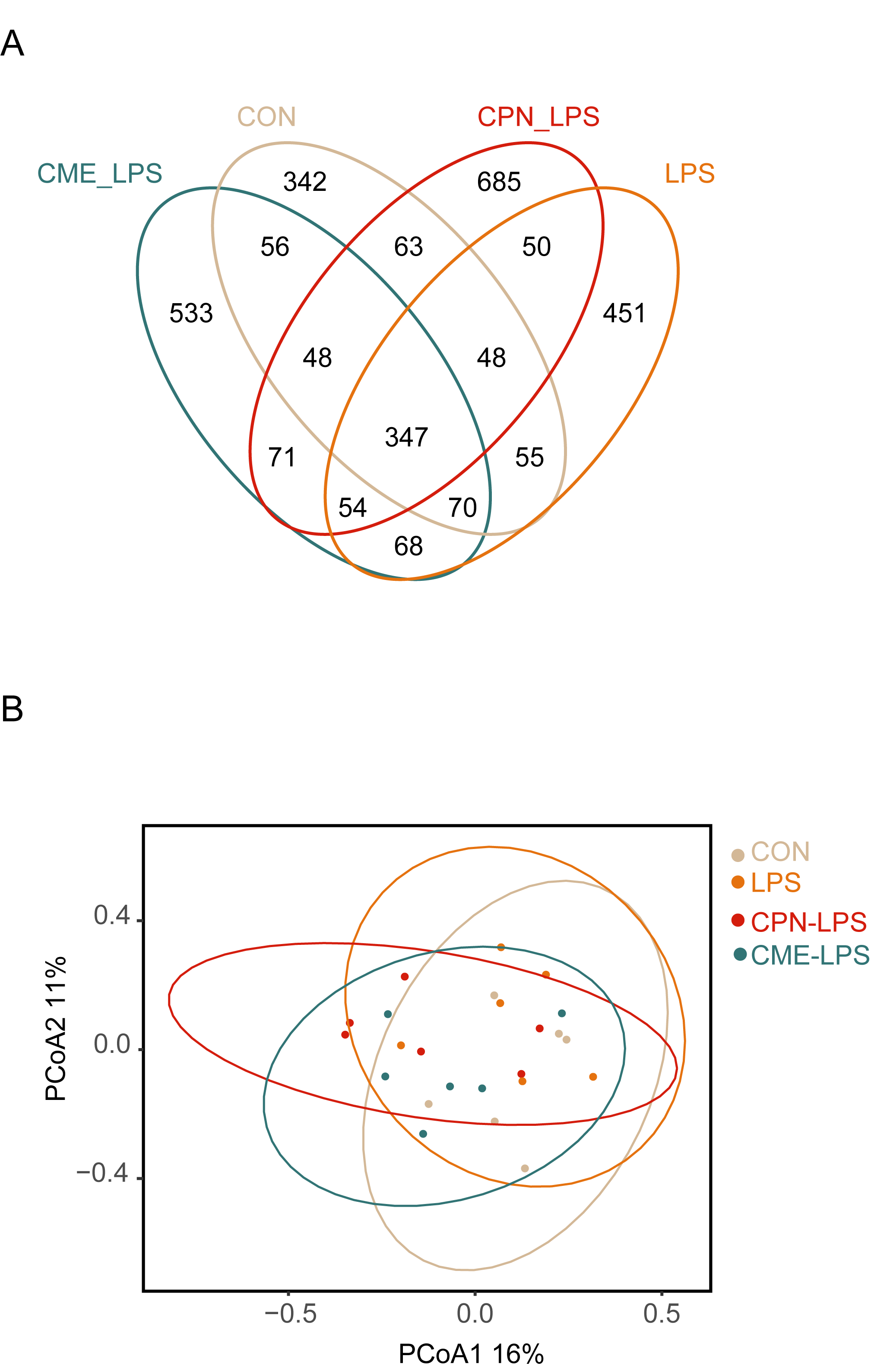


**Fig. S3**

**
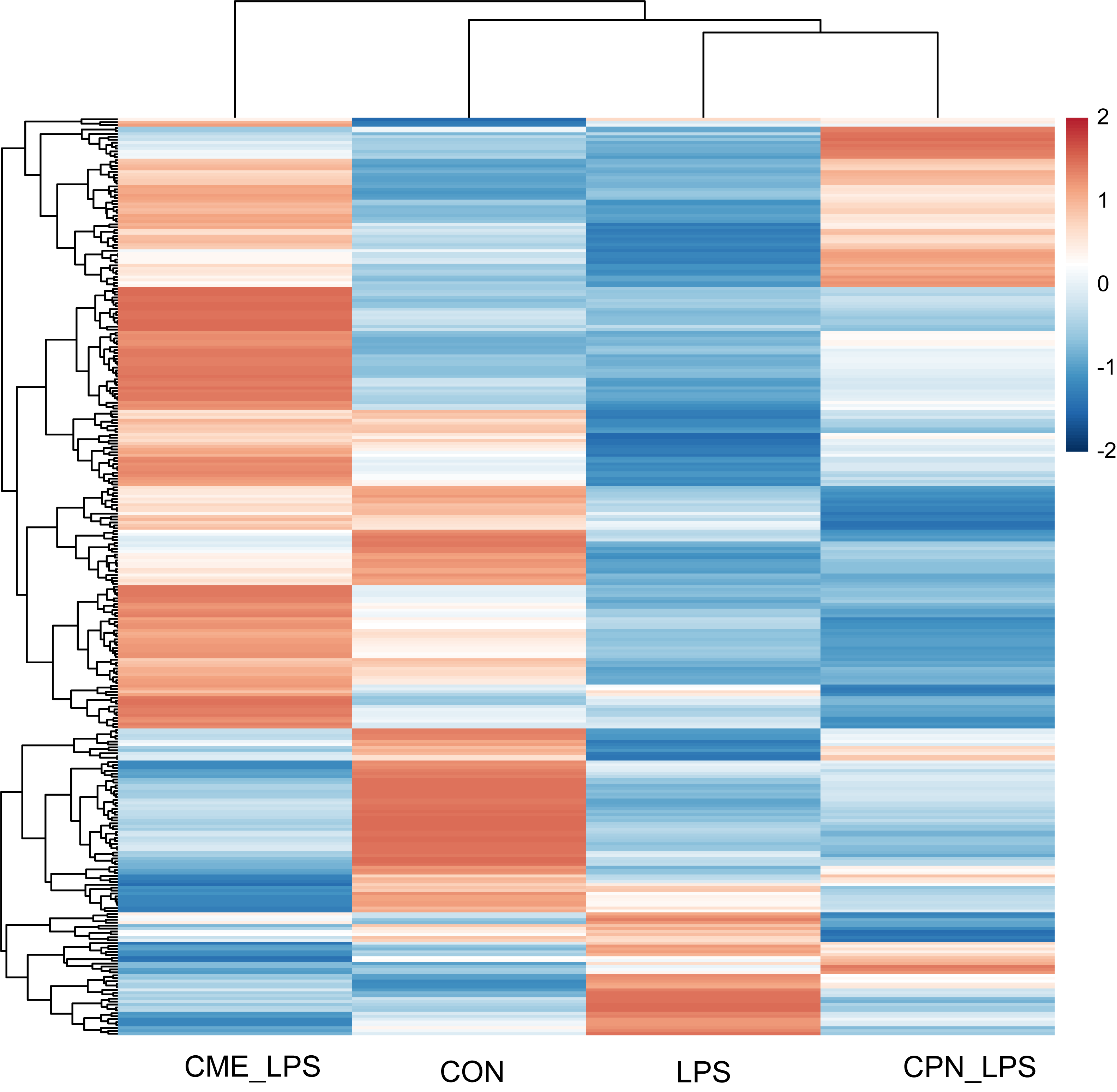
**
